# Safety and feasibility of pressurized intraperitoneal aerosol chemotherapy (PIPAC) associated with systemic chemotherapy: an innovative approach to treat peritoneal carcinomatosis

**DOI:** 10.1186/s12957-016-0892-7

**Published:** 2016-04-29

**Authors:** Manuela Robella, Marco Vaira, Michele De Simone

**Affiliations:** Unit of Surgical Oncology, Candiolo Cancer Institute-FPO, IRCCS, Strada Provinciale 142, km 3.95, 10060 Candiolo, TO Italy

**Keywords:** Colorectal cancer, Gastric cancer, Metastasis, Peritoneum, Pneumoperitoneum, Surgery

## Abstract

**Background:**

Pressurized intraperitoneal aerosol chemotherapy (PIPAC) is a new treatment that applies chemotherapeutic drugs into the peritoneal cavity as an aerosol under pressure. It improves local bioavailability of chemotherapeutic drugs as compared with conventional intraperitoneal chemotherapy. It has been proved to be safe and feasible if performed as an exclusive treatment in patients affected by peritoneal carcinomatosis. The first results in patients treated with PIPAC associated with systemic chemotherapy are presented.

**Methods:**

Between June 2015 and February 2016, 57 PIPAC applications with oxaliplatin or cisplatin + doxorubicin every 6 weeks at 37 °C and 12 mmHg for 30 min were performed. Forty PIPAC procedures performed in 14 patients were included in this study; thirteen patients were undergoing systemic chemotherapy with a wash-out interval of at least 2 weeks before and 1 week after each PIPAC. Safety, tolerability, and postoperative complications were assessed by collection of adverse events according to the Common Terminology Criteria for Adverse Events (CTCAE) 2.

**Results:**

Forty PIPAC administrations were performed in 14 patients with no major perioperative complications. CTCAE grades 1 and 2 were observed after six and eight procedures, respectively, for abdominal pain and nausea. Renal and hepatic functions were not impaired; no cumulative renal toxicity was observed after repeated PIPAC procedures in association with systemic chemotherapy.

**Conclusions:**

These preliminary data show that the association of PIPAC and systemic chemotherapy does not induce significant hepatic and renal toxicity. It allows inclusion of patients with extraperitoneal disease or at a high risk of developing it. Further studies are needed to assess whether this combination therapy could become part of the standard treatment for peritoneal carcinomatosis.

## Background

For a long time, peritoneal carcinomatosis (PC) has been regarded as a terminal disease. Traditional treatment consists of systemic chemotherapy, with or without palliative surgery, resulting in poor effects in terms of outcome. With the development of more effective chemotherapeutic drugs and target therapies, expected median survival rose to 20 months for colorectal cancer (CRC) [1], 4–10 months for epithelial ovarian cancer (EOC) [2], 7–10 months for gastric cancer (GC) [3], and 6–12 months for peritoneal mesothelioma (DMPM) [4]. Sugarbaker et al. challenged this oncologic philosophy and suggested that PC, in the absence of distant metastases, should be considered as a locally advanced disease for which an aggressive approach can be justified [5]. For these reasons, a multimodal treatment based on cytoreductive surgery (CRS) and hyperthermic intraperitoneal chemotherapy (HIPEC) in association with systemic therapy was developed.

Unfortunately, only selected patients may undergo this combined procedure, and anyway, its role in the treatment of some pathologies such as gastric cancer remains a matter of considerable debate [6]. Moreover, this treatment is still hampered by significant risks and side effects with a 30-day mortality rate of 5 % in referral centers [7].

A further aspect of intraperitoneal chemotherapy is the pharmacological limitation in terms of poor drug distribution in the peritoneal cavity and penetration into peritoneal nodules. Pressurized intraperitoneal aerosol chemotherapy is an innovative intraperitoneal chemotherapy (IPC) concept that seems to enhance the effectivity of IPC by taking advantage of the physical properties of gas and pressure [8]. Preliminary experiences reported in literature are based on strict exclusion criteria: patients with extra abdominal metastatic disease including retroperitoneal disease, patients who underwent chemotherapy or surgery within the last 4 weeks, or patients who undergo any cancer-specific treatment during the study cannot be included [9–12]. The narrowness of the selection criteria may exclude many patients that, presenting an advanced stage of the disease, often show extra-abdominal lesions or simply aortic/para-aortic lymph node recurrence.

PIPAC procedure is resulted to be safe, with no postoperative renal and hepatic toxicity [13–15]; in this paper, we would prove, through our preliminary results, the safety and feasibility of PIPAC associated with systemic chemotherapy in order to expand the cohort of patients who can most benefit from this treatment.

## Methods

Between June 2015 and February 2016, 57 PIPAC procedures were carried out in 29 patients with PC (seven CRC, five EOC, four DMPM, two appendiceal cancers, nine GC, two breast cancers); we considered for this study 40 applications performed in 14 patients (two CRC, three EOC, two DMPM, one appendiceal cancer, six GC); some of them were treated within the framework of off-label use applications, awaiting for Italian Drug Agency (AIFA—Agenzia Italiana del Farmaco) approval of an open-label, single-arm, phase II clinical trial [https://clinicaltrials.gov/ct2/show/NCT02604784]. Patients aged between 18 and 78 years, presenting a good performance status (ECOG ≤ 2) with clinical and pathological confirmation of peritoneal carcinomatosis from gastric, colorectal and ovarian cancers, or primary peritoneal tumors were included. Patients were required to have undergone at least one line of previous i.v. standard chemotherapy in DMPM and two lines in CRC and EOC. Patients, contrary to other trials, were not required to stop any other cancer-specific treatment during our study.

Specifically, we included patients undergoing systemic chemotherapy but had stopped it during 2 weeks before and 1 week after PIPAC procedure. All patients signed an informed consent. Lastly, patients were eligible if they had blood and electrolyte counts, liver, renal, and cardiopulmonary function parameters within 10 % of the normal range; tumor mass present on CT-scan in order to allow tumor response assessment with RECIST-criteria.

Finally, any of the following was regarded as a criterion for exclusion from the study: bowel obstruction, severe renal impairment, myelosuppression, severe hepatic impairment, severe myocardial insufficiency, recent myocardial infarction, severe arrhythmias, immuno-deficiency such as patients with an immunosuppressive medication or a known immune system disease, creatinine clearance <60 ml /min, pregnancy, previous treatment reaching the maximum cumulative dose of doxorubicin, daunorubicin, epirubicin, idarubicin, and/or other anthracyclines and anthracenediones, known allergy to cisplatin or other platinum-containing compounds or to doxorubicin, refusal to conduct complete abstinence from heterosexual relationships or agree to use an effective clinically acceptable method (with failure rate <1 %) during the study and the following 6 months after the last treatment.

All operations were performed under general anesthesia; venous thromboembolism prophylaxis was administered the night before surgery using low molecular weight heparin (LMWHs), and antibiotic prophylaxis with a single dose of Cefazolin 2 g IV was administered 30 min before surgery.

An open access with a midline 5–6-cm incision was performed and a single-port platform (Quad-Port Olympus) was positioned according to our original technique. A 12 mmHg CO2 pneumoperitoneum was inflated. Ascites were removed if present and the amount documented. PC extent was evaluated according to the Sugarbaker Peritoneal Cancer Index (PCI) [16], and multiple peritoneal biopsies were taken. A nebulizer (MIP, Reger Medizintechnik, Rottweil, Germany) was connected to an high-pressure injector and inserted into the peritoneal cavity; the tightness of the abdomen was documented with a CO_2_ zero-flow. The camera and the nebulizer are maintained in position by a self-retaining retractor (Thompson). A pressurized aerosol containing cisplatin 7.5 mg/m2 body surface in 150 ml NaCl 0.9 % + doxorubicin 1.5 mg/m2 body surface in 50 ml NaCl 0.9 % in patients with EOC and DMPM and oxaliplatin 92 mg/m2 body surface in 150 ml dextrose solution in patients with CRC was applied through the nebulizer. Injection parameters were flow of 30 ml/min and a max upstream pressure of 200 psi with an intra-abdominal pressure of 12 mmHg [10–12]. The injection was remote-controlled in order to avoid occupational exposure. The capnoperitoneum was then maintained for 30 min at 37 °C. At the end, the aerosol was exsufflated through two sequential micro-particle filters into the air-waste system of the hospital. Single-port platform was removed; no abdominal drain tube was applied. Nasogastric tube and urinary catheter were removed at the end of the operation.

Peripheral venous blood was collected preoperatively, the day of the intervention and daily until the discharge. Creatinine and urea clearances measurement was performed before each operation.

Data of all patients who underwent PIPAC procedure were included in a prospectively maintained database. Safety, tolerability, and postoperative complications were assessed by collection of adverse events, according to the Common Terminology Criteria for Adverse Events (CTCAE) 2 including physical examination results and laboratory assessments (chemistry and hematology).

Statistics was performed using SPSS version 22 software. Comparative statistics over time was performed by one-way repeated analysis of quantitative variables (*t* student test).

## Results

Between June 2015 and February 2016, 40 PIPAC procedures were performed in five patients (one DMPM, one EOC, one CRC, one appendiceal cancer, one GC); in 13 patients, PIPAC was performed in association with systemic chemotherapy with a wash-out interval of at least 2 weeks before and 1 week after each procedure. Patient characteristics including the kind of systemic chemotherapy performed are shown in Table 1.

**Table 1:** Patient Characteristics

	Age	Disease	First diagnosis	Previous surgery	Previous sCT	Associated sCT	PIPAC procedure
Pat 1	62	DMPM	April 2012	2 CRS + HIPEC	1 line	–	2
Pat 2	71	EOC	September 2010	ARR, ovarectomy, hysterectomy, omentectomy, lymphadenectomy	7 lines	Topotecan (days 1, 8, 15)	3
Pat 3	68	CRC	May 2014	Explorative laparotomy	2 lines	Folfox + Cetuximab	4
Pat 4	43	PMP	August 2013	Debulking	2 lines + irinotecan IP	Folfoxiri	4
Pat 5	61	EOC	September 2012	ARR, small bowel resection, ovarectomy, hysterectomy, omentectomy	3 lines	Weekly paclitaxel	3
Pat 6	39	GC	December 2014	Explorative laparotomy	2 lines	Folfiri	3
Pat 7	51	GC	November 2013	Gastric resection	2 lines	Paclitaxel + ramucirumab	3
Pat 8	47	GC	July 2015	Explorative laparoscopy	1 line	Xelox	3
Pat 9	53	EOC	September 2014	Ovarectomy, hysterectomy,	2 lines	Paclitaxel	2
Pat 10	78	DMPM	April 2015	Explorative laparoscopy	1 line	Pemetrexed	3
Pat 11	51	CRC	August 2011	CRS + HIPEC	2 lines	Cetuximab	2
Pat 12	55	GC	January 2015	Gastric resection, omentectomy,	1 line	CDDP + teysuno	2
Pat 13	45	GC	December 2013	Gastric resection	1 line	CDDP + gemcitabine	3
Pat 14	52	GC	July 2015		1 line	Paclitaxel + ramucirumab	3

*sCT* systemic chemotherapy, *CRS* cytoreductive surgery, *ARR* anterior rectal resection, *IP* intraperitoneal, *DMPM* diffuse malignant peritoneal mesothelioma, *EOC* epithelial ovarian cancer, *CRC* colorectal cancer, *PMP* pseudomyxoma peritonei, and *GC* gastric cancer

We reported a mean operative time of 86 min (range 45–145), a laparoscopic access rate of 100 % with no postoperative re-laparotomies. The mean hospital stay was 3 days. Mean PCI was 17 (range 12–21). CTCAE grades 1 and 2 were observed in six and eight patients, respectively. Six out of 13 patients presented mild abdominal pain and eight patients complained of nausea. None presented fever. No postoperative mortality was reported. Patient 1 received only two PIPAC procedures because of the inability to create a good laparoscopic chamber due to adhesion increase.

A slight leukocytosis was recorded after most of the procedures, often associated with an increase in C-reactive protein (CRP) because of the chemical peritonitis due to the chemotherapy agents with a peak on the second postoperative day (POD) (mean 0.048 ± 0.036, *p* = 0.002) followed by a decrease on POD 3.

**Fig. 1:**
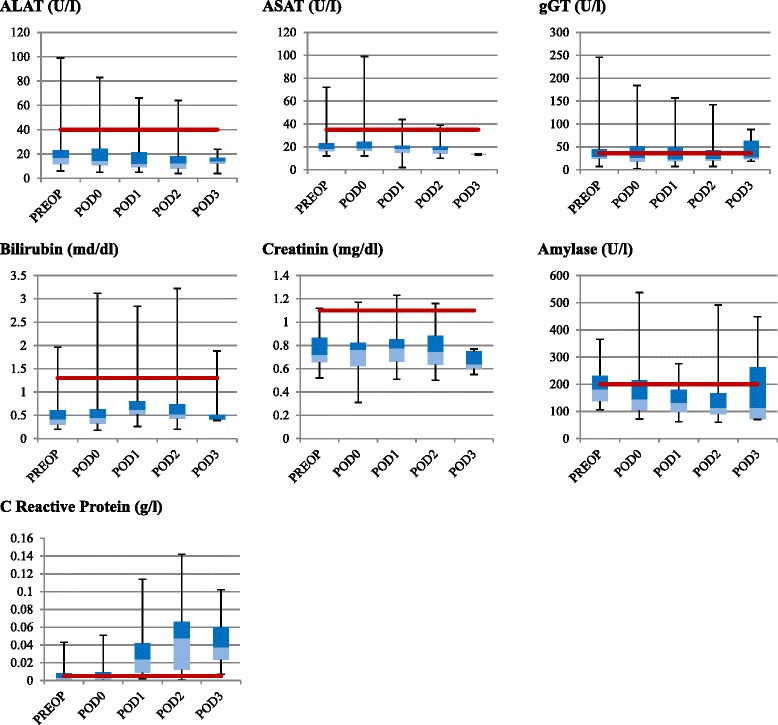
Box plot of liver, renal, and pancreatic functions before the intervention and during the hospital stay. The *light blue and blue boxes* represent the second and the third quartile, respectively. The *upper and lower whiskers* represent scores outside the middle 50 %, the highest and lowest value, respectively. The *red line* represents the upper limit of normal range of measured parameters. *PIPAC* pressurized intraperitoneal aerosol chemotherapy, *CTCAE* common terminology criteria for adverse events, *PC* peritoneal carcinomatosis, *CRC* colorectal cancer, *EOC* epithelial ovarian cancer, *GC* gastric cancer, *DMPM* diffuse malignant peritoneal mesothelioma, *CRS* cytoreductive surgery, *HIPEC* hyperthermic intraperitoneal chemotherapy, *IPC* intraperitoneal chemotherapy, *CT* computed tomography, *LMWH* low molecular weight heparin, *PCI* Peritoneal Cancer Index, *CRP* C-reactive protein, *POD* postoperative day, and *QoL* quality of life

No signs of liver toxicity were observed after the procedures, and all indices of liver function remained in the normal range; we reported a minimum increase of ASAT on POD 0 (mean 24 ± 15.3, *p* = 0.21); ALAT and serum gamma-GT remain stable during the whole hospital stay. Analogously, serum amylase values were stable, with a peak on POD 3 (mean 193.5 ± 145.5), not significantly higher than the preoperative value (mean 193.0 ± 66.6). Total bilirubin serum levels reported a slight increase on POD 1 (mean 0.8 ± 0.6) without any clinical relevance. Renal function was not impaired; preoperative serum creatinine and creatinine/urea clearances were in range and did not increase; preoperatively mean serum creatinine value was 0.8 ± 0.2 and it remained stable during the whole hospital stay. No cumulative renal toxicity was observed after repeated PIPAC procedure at 6-week intervals in association with systemic and intraperitoneal chemotherapy. The details about liver and renal toxicity laboratory data are reported in Fig. 1.

At discharge, each patient presented good general conditions, blood tests were in range, and regularly underwent the planned systemic chemotherapy without cumulative toxicity.

Quality of life (QoL) was recorded routinely in all patients before the enrollment and after each PIPAC procedure through two questionnaires: SF-36 and EORTC QLQ-30. No further deterioration of physical, emotional, and cognitive scores during therapy were recorded.

## Discussion

These preliminary data about patients treated with PIPAC in association with systemic chemotherapy show that the combined treatment does not induce significant hepatic and renal toxicity.

PIPAC pharmacokinetics permit to use a minimal drug dose reaching a higher intraperitoneal concentration than in HIPEC; in fact, intra-abdominal pressure increases tissue uptake, intra-tumoral drug concentration [17, 18], and the micronization of the cytostatic agent creates a thin film of microdroplets over the entire peritoneal cavity, increasing the contact surface area between drugs and tissues. In fact, the micro-injection pump creates micron-size drug particles reducing the average diameter of a chemotherapeutic infusion.

On the basis of these features, PIPAC resulted to be a well-tolerated treatment without major postoperative complications. Liver and renal tests showed neither acute nor cumulative toxicity after the procedures. Objective, radiological, and serological disease regression was observed in five patients; stable disease was recorded in two patients. In seven patients, we reported a disease progression at a later stage. The aim of this paper is to prove the safety and feasibility of PIPAC associated with systemic chemotherapy; this demonstration, in fact, may allow treatment of patients who, presenting an advanced stage of disease, may have ascites and complain of sub-occlusive symptoms and abdominal pain due to peritoneal disease. The complementary systemic treatment permits to include patients who, for cancer clinical stage, may present retroperitoneal adenopathy, parenchymal metastases, or even extra-abdominal disease, for which PIPAC is not effective, expanding the population of patients who can most benefit from this treatment.

In patients presenting a good general condition but a worsening quality of life because of peritoneal disease diffusion, the combination of the two treatments enables rapid symptom palliation with PIPAC and a risk reduction of extra-abdominal metastasis thanks to systemic chemotherapy.

The progression disease reported in five patients probably means that the PIPAC procedure has yet to be improved. Probably, PIPAC may enhance the activity of systemic chemotherapy (for example, by reducing the intra-tumoral interstitial fluid pressure), but as PIPAC alone, on the basis of our preliminary experience with the current drugs doses, it is not sufficiently effective. A dose-finding study for the determination of the optimal dose is mandatory.

In this perspective, PIPAC may not only be considered a palliative treatment, but in combination with systemic chemotherapy, with appropriate drug doses, it could possibly become part of the standard therapeutic course of peritoneal carcinomatosis, as it has been shown for IPC (intraperitoneal chemotherapy) [19, 20] and HIPEC for certain diseases [21–34].

## Conclusions

This preliminary analysis demonstrates that the combined treatment based on PIPAC and systemic chemotherapy does not induce significant hepatic and renal toxicity.

Certain tumors in advanced stage of disease, may present not only PC but also lymph node or retroperitoneal metastasis. In those cases, PIPAC would be ineffective, but considering its low toxicity and postoperative morbidity, may be associated with systemic chemotherapy. This combined treatment as well as being ethically accepted, may be a useful strategy for patients presenting extraperitoneal disease or at a high risk of developing it.

These preliminary results obtained in a small cohort of patients provide a rational for prospective studies in order to improve the technique and assess whether this combination therapy could become part of the standard treatment for peritoneal carcinomatosis.
